# Measuring the Rate of Information Exchange in Point-Process Data With Application to Cardiovascular Variability

**DOI:** 10.3389/fnetp.2021.765332

**Published:** 2022-01-28

**Authors:** Gorana Mijatovic, Riccardo Pernice, Alessio Perinelli, Yuri Antonacci, Alessandro Busacca, Michal Javorka, Leonardo Ricci, Luca Faes

**Affiliations:** ^1^ Faculty of Technical Science, University of Novi Sad, Novi Sad, Serbia; ^2^ Department of Engineering, University of Palermo, Palermo, Italy; ^3^ CIMeC, Center for Mind/Brain Sciences, University of Trento, Rovereto, Italy; ^4^ Department of Physics and Chemistry “Emilio Segrè,” University of Palermo, Palermo, Italy; ^5^ Department of Physiology and Biomedical Center Martin, Jessenius Faculty of Medicine, Comenius University, Martin, Slovakia; ^6^ Department of Physics, University of Trento, Trento, Italy

**Keywords:** information dynamics, point processes, mutual information rate, heart rate variability, cardiovascular time series

## Abstract

The amount of information exchanged per unit of time between two dynamic processes is an important concept for the analysis of complex systems. Theoretical formulations and data-efficient estimators have been recently introduced for this quantity, known as the mutual information rate (MIR), allowing its continuous-time computation for event-based data sets measured as realizations of coupled point processes. This work presents the implementation of MIR for point process applications in Network Physiology and cardiovascular variability, which typically feature short and noisy experimental time series. We assess the bias of MIR estimated for uncoupled point processes in the frame of surrogate data, and we compensate it by introducing a corrected MIR (cMIR) measure designed to return zero values when the two processes do not exchange information. The method is first tested extensively in synthetic point processes including a physiologically-based model of the heartbeat dynamics and the blood pressure propagation times, where we show the ability of cMIR to compensate the negative bias of MIR and return statistically significant values even for weakly coupled processes. The method is then assessed in real point-process data measured from healthy subjects during different physiological conditions, showing that cMIR between heartbeat and pressure propagation times increases significantly during postural stress, though not during mental stress. These results document that cMIR reflects physiological mechanisms of cardiovascular variability related to the joint neural autonomic modulation of heart rate and arterial compliance.

## 1 Introduction

The mutual information (MI) between two random variables is a central concept in information theory. MI is an important quantity with huge practical relevance, as it quantifies how much information is exchanged between two complex systems or is shared by two data sets. Indeed, thanks to these characteristics, MI is ubiquitously employed in diverse fields of science and engineering to assess linear and non-linear interactions, e.g., between electronic oscillators (Minati et al., 2018), financial systems (Fiedor, 2014), climatological variables (Perinelli et al., 2021), brain units (Mijatovic et al., 2021b) or physiological systems (Valderas et al., 2019). In all these application fields, the study of dynamical systems, i.e., systems whose state evolves over time, is central to the understanding of the underlying phenomena. Therefore, dynamic formulations of MI in which the observed variables are associated with temporal information are recommended for a proper assessment of the interactions between the system units. In this study we consider the MI rate (MIR), a well-known quantity measuring the amount of information shared by two random processes per unit of time (Duncan, 1970). In particular, we focus on the computation of MIR for point processes, i.e., processes where the relevant information stands in the times of occurrence of specific events. This class of processes is widely adopted in neuroscience, for instance to study the spiking activity of neural populations acquired through multi-electrode recording techniques (Truccolo et al., 2005), and in the field of cardiovascular variability, where the point process nature of the human heartbeats has inspired the development of event-based models to describe the heart rate and its interaction with vascular, respiratory and metabolic variables (Barbieri et al., 2005; Valenza et al., 2018).

The calculation of dynamic information measures, such as the MIR or the transfer entropy rate (TER) quantifying the rate of directed (causal) information flow between stochastic processes (Schreiber, 2000; Spinney et al., 2017) is well-established for discrete-time processes, i.e., processes defined at discrete time instant, which represent the sampling rate of continuous-time signals or the rate of a physiological oscillator (e.g., the cardiac pacemaker); in this context, a number of practical approaches exist to provide data-efficient estimates (Vicente et al., 2011; Faes et al., 2015). On the other hand, the definition and practical computation of these measures for continuous-time processes defined at each time instant with arbitrarily small resolution, and more specifically for point processes, is much more cumbersome. The classical way to compute MIR and TER for point process or other event-based data typically relies on binning of the temporal axis followed by the application of discrete-time estimators (Pasquale et al., 2008), but unavoidably implies loss of information and strong dependence on the parameters related to time discretization (Mijatovic et al., 2021a; Shorten et al., 2021). Only recently, the theoretical formalism (Spinney et al., 2017; Spinney and Lizier, 2018) and the design of estimation approaches for the TER (Shorten et al., 2021) and MIR (Mijatovic et al., 2021a) has been introduced in the context of neuroscience applications. In particular, Mijatovic et al. (2021a) have shown that for point process data the MIR can be expressed as the sum of the TER computed along the two directions of interaction between the two analyzed processes, and have exploited the TER estimation methods introduced by (Shorten et al., 2021) to design a data-efficient estimator of the MIR for coupled point processes. These works are of a great practical relevance, because they open the way for a reliable non-parametric, continuous-time estimation of the information transfer for event-based processes.

In this work, we exploit the MIR estimator introduced in (Mijatovic et al., 2021a) to assess the rate of information shared between cardiovascular point processes. Specifically, we focus on cardiovascular interactions assessed between the cardiac pacemaker, studied by the heartbeat timings and measured from the ECG, and the times of arrival to the body periphery of the sphygmic wave, measured through finger photoplethysmography. The application of event-based frameworks to heartbeat and pulse arrival times entertains a different perspective on the study of cardiovascular regulation than more classical analyses performed on time series of the heart period and arterial pressure variability (Cohen and Taylor, 2002; Porta and Faes, 2015), and leads to address related but different physiological mechanisms. In particular, while classical time series analysis methods investigate cardiovascular interactions focusing on baroreflex regulation and mechanical mechanisms (Faes et al., 2013; Javorka et al., 2017), the study of coupled point processes may reveal the physiological mechanisms that modulate the arterial pressure, the contractility of the ventricles and vasomotion (Okada et al., 1996; Chan et al., 2007). Since these mechanisms typically operate on short time scales involving a few heartbeats, and due to stationarity issues, the analysis of these processes is typically restricted to short realizations (few hundred events). A practical consequence of this restriction is the difficulty of obtaining reliable estimates in the presence of short series of data. To test the applicability of the MIR estimator on short realizations of point process data, we assess the estimation bias in simulations of uncoupled point processes generated for different parametric probability distributions. When there is no coupling between the processes, a positive bias can be misinterpreted as a weak coupling, while a negative bias makes a non-negative measure like the MIR of difficult interpretation. We provide a solution to this problem, by modifying the MIR estimator and introducing a corrected MIR (cMIR) measure for which the bias is reduced; the correction employs surrogate time series, which reproduce the bias occurring for uncoupled point processes. The novel cMIR measure is tested first in simulated point process models that reproduce the coupled occurrence of the heartbeat times and of the arrival instants of the blood pressure wave at the body periphery, and then in real point process series measured from healthy subjects monitored in resting state and during postural and mental stress (Javorka et al., 2017).

## 2 Information-Theoretic Measures to Assess the Dynamic Interaction Between Stochastic Processes

This section presents the mathematical background necessary to assess the information shared between continuous-time stochastic processes. Information-theoretic measures are typically employed to treat dynamic systems in discrete time, i.e. systems can be described by processes whose states are mapped by times series values. However, many theoretical and real-world systems are naturally described by processes defined in continuous time, whose available discrete-time signals represent approximate realizations. The most accurate information-theoretic treatment of continuous-time processes is that using random functions in place of collections of random variables to quantify information dynamics (Spinney et al., 2017). In the following subsections, we show how to employ random functions to define the information dynamically shared between two continuous-time processes, how to express it in terms of the information transferred along the two directions of interaction between the processes, and how to formalize its computation and practical estimation in the particular case of point processes.

### 2.1 Mutual Information and Transfer Entropy Rates

Let us consider two possibly coupled dynamical systems X and Y such that their evolution over time is mapped by the continuous-time stochastic processes *X* = {*X*_*t*_} and *Y* = {*Y*_*t*_}, which are defined at each continuous-time instant t∈R . A well-known undirected measure of the dynamical interaction between *X* and *Y* is the *mutual information rate* (MIR), which quantifies the amount of information exchanged per unit of time by the two processes (Duncan, 1970). If the processes are stationary, the MIR is defined as

$${\dot{I}}_{X;Y}=\underset{\tau \to \infty }{\lim}\frac{1}{\tau }I\left(X_{t-\tau :t};Y_{t-\tau :t}\right),$$ (1)

where *I* (⋅; ⋅) denotes mutual information (MI) and *τ* is the duration of the temporal window over which the MI is computed. The notation *X*_*t*−*τ*:*t*_ denotes the random function expressing the stochastic process evaluated along the time interval of duration *τ* ending at the time *t*, also referred to as *path* (Spinney et al., 2017), i.e. *X*_*t*−*τ*:*t*_ = {*X*_*s*_ : *t* − *τ* ≤ *s* < *t*} (the same holds for the process *Y*); note that the MI in Eq. 1, and consequently the MIR, are independent on *t* due to stationarity.

The MIR defined above, as any other information measure applied to continuous-time processes, cannot be readily formulated in terms of the probability mass functions or densities used for discrete and continuous random variables. In continuous time, a viable approach is to establish a generalized form for the information measures via measure-theoretic approaches that unify under one framework the methods specifically developed for discrete and continuous random variables (Spinney et al., 2017). In this framework, information measures can be expressed by using the Radon-Nykodim derivative between appropriate random density functions defined on paths in place of the ratio between probability distributions of random variables adopted in discrete-time (Gray, 2011). The MI measure in Eq. 1 can be then expressed in a generalized form as (Duncan, 1970)

$$I\left(X_{t-\tau :t};Y_{t-\tau :t}\right)=E_{P}\left[\ln\frac{dP\left[x_{t-\tau :t}|y_{t-\tau :t}\right]}{dP\left[x_{t-\tau :t}\right]}\right],$$ (2)

where the expectation is taken over the path realizations *x*_*t*−*τ*:*t*_ and *y*_*t*−*τ*:*t*_ of the random functions *X*_*t*−*τ*:*t*_ and *X*_*t*−*τ*:*t*_, and the argument of the logarithm is the Radon-Nykodim derivative of two probability measures defined on path functions. With a similar formalism, Spinney and colleagues have formalized different measures of information dynamics for continuous-time processes (Spinney et al., 2017; Spinney and Lizier, 2018). In particular, the transfer entropy rate (TER) from the ‘source’ process *Y* to the ‘target’ process *X* is defined as (Spinney et al., 2017)

$${\dot{T}}_{Y\to X}\left(t,\tau \right)=\underset{\Delta t\to 0}{\lim}\frac{1}{\Delta t}T_{Y\to X}\left(t,\Delta t,\tau \right),$$ (3)

where

$$T_{Y\to X}\left(t,\Delta t,\tau \right)=E_{P}\left[\ln\frac{dP\left[x_{t+\Delta t}|x_{t-\tau :t},y_{t-\tau :t}\right]}{dP\left[x_{t+\Delta t}|x_{t-\tau :t}\right]}\right]$$ (4)

is the transfer entropy (TE) formulated in terms of a Radon-Nykodim derivative of conditional probability measures similarly as in Eq. 2 for the MI, and the normalization by the time interval Δ*t* ensures convergence of the TER in the limit of small Δ*t* (Spinney et al., 2017). For stationary processes *X* and *Y*, the TER is independent on the time *t*; moreover, considering realizations of infinite duration yields the constant TER measure

$${\dot{T}}_{Y\to X}=\underset{\tau \to \infty }{\lim}{\dot{T}}_{Y\to X}\left(t,\tau \right),$$ (5)

which quantifies the rate of information transferred along the causal direction from *Y* to *X*. By reversing the role of the two processes, the information transferred along the opposite causal direction can be quantified by the TER measure ${\dot{T}}_{X\to Y}$ .

The measures of the rates of information exchanged by *X* and *Y* defined in Eqs. 1, 5 are related to each other by a decomposition that expresses the MIR between *X* and *Y* as the sum of the TER along the two directions *X* → *Y* and *Y* → *X*, plus a term related to the instantaneous interaction between the two processes. Specifically, by using information-theoretic rules on Eq. 2 and recognizing Eq. 4 as a conditional MI, i.e., *T*_*Y*→*X*_ (*t*, Δ*t*, *τ*) = *I* (*X*_*t*+Δ*t*_; *Y*_*t*−*τ*:*t*_|*X*_*t*−*τ*:*t*_), the MIR can be expanded as

$${\dot{I}}_{X;Y}={\dot{T}}_{X\to Y}+{\dot{T}}_{Y\to X}+{\dot{I}}_{X;Y}^{0},$$ (6)

where the term

$${\dot{I}}_{X;Y}^{0}=\underset{\Delta t\to 0}{\lim}\underset{\tau \to \infty }{\lim}\frac{1}{\Delta t}I\left(X_{t+\Delta t};X_{t+\Delta t}|X_{t-\tau :t},Y_{t-\tau :t}\right)$$ (7)

quantifies the rate of information instantaneously exchanged between the two processes conditioned to the knowledge of their past histories. The derivation of the important relation Eq. 6, where all three terms are quantified in [nats/s], is reported in the Appendix.

### 2.2 Computation for Bivariate Point Processes

In this subsection we formulate the computation of MIR for point processes. A point process is a particular class of continuous-time process that is uniquely characterized by a series of indistinguishable events described by their time of occurrence. In a bivariate context, the statistical description of two point processes is provided in terms of the instants marking the event times, i.e., by writing *X* = {*x*_*i*_}, *i* = 1, … , *N*_*X*_, and *Y* = {*y*_*j*_}, *j* = 1, … , *N*_*Y*_, where *x*_*i*_ and *y*_*j*_ represent the times of the *i*th event in *X* and of the *j*th event in *Y*, respectively. For these point processes, the MIR can be computed by leveraging the decomposition provided in Eq. 6 (Mijatovic et al., 2021a) and making the assumption that simultaneous events are not possible, i. e, *x*_*i*_ ≠ *y*_*j*_, *∀i* = 1, … , *N*_*X*_, *j* = 1, … , *N*_*Y*_ (Spinney et al., 2017; Mijatovic et al., 2021a; Shorten et al., 2021). This assumption implies that the measure ${\dot{I}}_{X;Y}^{0}$ of instantaneous information exchange between *X* and *Y* defined in Eq. 7 is null, so that the MIR between two point processes simply becomes the sum of the two TER terms

$${\dot{I}}_{X;Y}={\dot{T}}_{X\to Y}+{\dot{T}}_{Y\to X}.$$ (8)

Starting from Eq. 8, the MIR can be calculated by employing methods to define (Spinney et al., 2017) and compute (Shorten et al., 2021) the TER for point processes. Specifically, the TER from *Y* to *X* is formulated as

$${\dot{T}}_{Y\to X}={\bar{\lambda }}_{X}E_{p_{x}}\left[\ln\frac{\lambda_{X,x_{i}|X_{x_{i}}^{-},Y_{x_{i}}^{-}}}{\lambda_{X,x_{i}|X_{x_{i}}^{-}}}\right],$$ (9)

where ${\bar{\lambda }}_{X}=N_{X}/T$ is the average event rate of *X*, *N*_*X*_ is the number of target events, and *T* is the duration of the target process; in Eq. 9, $\lambda_{X,x_{i}|X_{x_{i}}^{-}}$ and $\lambda_{X,x_{i}|X_{x_{i}}^{-},Y_{x_{i}}^{-}}$ are the instantaneous event rates of the target process *X* evaluated at the time of its *i*th event *x*_*i*_, respectively conditioned on the history of *X* and on the histories of both *X* and *Y*. In general, the unconditioned instantaneous event rate of the process *X*, evaluated at the arbitrary time *u*, is given by $\lambda_{X,u}=\lim_{\Delta u\to 0}p_{u}\left(N_{X,u+\Delta u}-N_{X,u}=1\right)/\Delta u$ , where *N*_*X*_(*u*) is the counting process that returns the number of events occurred up to time *u*. At this point it is worth noting that, while the probability *p*_*u*_ is defined at any time point u∈R , the expectation in Eq. 9 is taken over the probability *p*_*x*_ of observing a quantity precisely at the time of target events *x*_*i*_, *i* = 1, … , *N*_*X*_ (Shorten et al., 2021). This important distinction, upon expressing the conditional event rates in terms of *p*_*u*_, making a Bayes inversion and noting that $\lim_{\Delta u\to 0}p_{u}\left(\cdot |N_{X,u+\Delta u}-N_{X,u}=1\right)=p_{x}\left(\cdot \right)$ , allows to reformulate the expression of the TER as (Shorten et al., 2021)

$${\dot{T}}_{Y\to X}={\bar{\lambda }}_{X}E_{p_{x}}\left[\ln\left(\frac{p_{x}\left(X_{x_{i}}^{-},Y_{x_{i}}^{-}\right)}{p_{u}\left(X_{x_{i}}^{-},Y_{x_{i}}^{-}\right)}\cdot \frac{p_{u}\left(X_{x_{i}}^{-}\right)}{p_{x}\left(X_{x_{i}}^{-}\right)}\right)\right].$$ (10)

Equation 10 shows that the TER depends on the probabilities of the process histories $X_{x_{i}}^{-}$ and $Y_{x_{i}}^{-}$ , evaluated at target events and at arbitrary time points (respectively, *p*_*x*_ and *p*_*u*_), whose statistical average is taken only at target events (i.e., over *p*_*x*_). The last expression constitutes the basis for the MIR estimation strategy presented in the next subsection.

### 2.3 Practical Estimation

The approach for MIR estimation, devised in (Mijatovic et al., 2021a; Shorten et al., 2021) and briefly presented in the following, relies on creating history embeddings that cover the past states of the two observed point processes, implementing an operational formulation of Eq. 10 to estimate the TER, and finally using Eq. 8 to obtain the MIR estimate.

**FIGURE 1:**
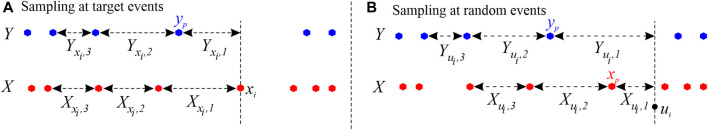
Example of history embeddings used to approximate the past states of a target point process *X* and a source process *Y* described by the event times depicted as red and blue dots, respectively. In this example, embeddings are reconstructed with an embedding length *l* = 3.

In the estimation of the TER from the source process *Y* to the target process *X*, the procedure for building history embeddings approximates the past history of the two processes observed either at the times of target events *x*_*i*_ = 1, … , *N*_*X*_, or at arbitrary time points *u*_*i*_ = 1, … , *N*_*U*_, sampled in continuous time. In the first case, illustrated in Figure 1A), the history embedding of the target *X* referred to the event *x*_*i*_ is approximated by taking *l* inter-event intervals, i.e., $X_{x_{i}}^{-}\approx X_{x_{i}}^{l}=\left\{x_{i-k+1}-x_{i-k},k=1,\ldots ,l\right\}$ ; the history embedding of the driver *Y* referred to *x*_*i*_ is approximated as $Y_{x_{i}}^{-}\approx Y_{x_{i}}^{l}=\left[x_{i}-y_{p},Y_{y_{p}}^{l-1}\right]$ , where *y*_*p*_ is the most recent driver event preceding *x*_*i*_. In the second case (Figure 1B), the histories of both processes as observed from *u*_*i*_ are approximated by taking the interval from the most recent event to *u*_*i*_ followed by *l* − 1 inter-event intervals, i.e., $X_{u_{i}}^{-}\approx X_{u_{i}}^{l}=\left[u_{i}-x_{p},X_{x_{p}}^{l-1}\right]$ , $Y_{u_{i}}^{-}\approx Y_{u_{i}}^{l}=\left[u_{i}-y_{p},Y_{y_{p}}^{l-1}\right]$ .

The history embeddings are then used to compute the entropy terms that compose the TER computed according to Eq. 10. Specifically, Eq. 10 can be expressed as

$${\hat{\dot{T}}}_{Y\to X}={\bar{\lambda }}_{X}\left[{\hat{H}}_{p_{u}}\left(X_{x_{i}}^{l},Y_{x_{i}}^{l}\right)-{\hat{H}}_{p_{x}}\left(X_{x_{i}}^{l},Y_{x_{i}}^{l}\right)+{\hat{H}}_{p_{x}}\left(X_{x_{i}}^{l}\right)-{\hat{H}}_{p_{u}}\left(X_{x_{i}}^{l}\right)\right],$$ (11)

where the estimates of the four entropies on the r.h.s. are obtained by approximating the past histories of infinite duration with the *l* − dimensional history embeddings, and computing the nearest neighbor entropy estimator (Vicente et al., 2011; Faes et al., 2015). Specifically, the terms ${\hat{H}}_{p_{x}}\left(\cdot \right)$ and ${\hat{H}}_{p_{u}}\left(\cdot \right)$ respectively refer to ‘standard’ differential entropy estimates where expectation is taken over the same probability distribution for which the log-likelihood is estimated, and to ‘cross-entropy’ estimates where the two distributions differ (a detailed procedure is given in Shorten et al. (2021); Mijatovic et al. (2021a)). The entropies are then estimated via the *k*NN estimator (Kozachenko and Leonenko, 1987), where the parameter *k* indicates the number of points used for searching the neighbors of each reference point; here, points are realizations of the history embeddings of dimension *l* or 2*l* specified in Eq. 11, and the search for neighbors is performed within the set of realizations taken at target events in the case of ‘standard’ entropy estimation, and within a set of realizations observed at arbitrary (randomly sampled) time points in the case of ‘cross-entropy’ estimation. The estimation algorithm, which is described in details in (Mijatovic et al., 2021a; Shorten et al., 2021), proceeds performing neighbor searches and range searches optimized to estimate together the four entropy terms in Eq. 11, in order to achieve compensation of the bias brought by the individual terms to the overall TER estimate. The TER from *X* to *Y* is estimated in the same way after reversing the role of the two point processes, and finally the MIR estimate is obtained by simply summing the two TER estimates in Eq. 8.

### 2.4 Corrected Measure of Mutual Information Rate

In this work, we face the issue of estimating the MIR from short realizations of coupled point processes. As any estimate of a measure computed on finite-length realizations of a process, the MIR exhibits bias and variance which typically depend on the system dynamics, the analysis parameters, and the time-series length. While the variance reflects random errors which cannot be corrected, the bias of an estimator is related to systematic errors that can be compensated by knowing the true value of the measure of interest and its average value computed over several repetitions of the analyzed process. However, unfortunately the true theoretical values are generally not known for the MIR of coupled point processes, as analytical results do not exist for the sampling distribution of kNN estimates of entropy quantities. Therefore, here we resort to an empirical procedure that follows previously proposed approaches using surrogate time series to reduce the bias of information-theoretic estimates (Marschinski and Kantz, 2002; Papana et al., 2011). Specifically, first we estimate the bias of the estimator computing its average over several realizations of uncoupled surrogate event series for which the expected MIR is zero, and then we use such average value to correct the MIR estimated on the original coupled processes. While this approach can be theoretically justified as a full correction of the bias only when the true coupling between the processes is zero, it has been shown to provide a reasonable compensation of the bias of coupling and causality measures even for coupled processes (Papana et al., 2011).

The correction procedure adopted in this work is based on the generation of surrogate time series that preserve the individual dynamics of a process while destroying any correlation between pairs of processes. While surrogates are typically used to set a significance threshold in the estimate of coupling measures (Faes et al., 2004; Lancaster et al., 2018), in our approach we do not apply a formal surrogate data test but rather correct the MIR for the bias estimated in the absence of coupling. To do this, after computing the MIR estimate ${\hat{\dot{I}}}_{X;Y}$ for a given realization of two point processes, we generate *M* surrogate point processes, estimate the MIR over each surrogate pair, and finally compute the corrected MIR (cMIR) as

$${\hat{\dot{I}}}_{X;Y}^{\left(c\right)}={\hat{\dot{I}}}_{X;Y}-{\hat{\dot{I}}}_{X;Y}^{\left(m\right)},$$ (12)

where ${\hat{\dot{I}}}_{X;Y}^{\left(m\right)}$ is the median of the MIR estimated over the *M* surrogate pairs; we use the median instead of the mean to consider possible deviations of the MIR values from a symmetric distribution. The use of the corrected measure Eq. 12 aims at reducing the bias of MIR in the case of absence of coupling between the two analyzed processes. To generate surrogate data, we adopted the procedure proposed by Shorten et al. (2021) in the context of TER estimation. This procedure implements a local permutation of the patterns forming the history embeddings for the two processes under the null hypothesis of independence of the present of the target and the history of the source given the history of the target. This null hypothesis is related to a more conservative test than that typically performed in TER/MIR estimation; while standard shuffling procedures destroy any relation between the current and past states of the target and the past states of the source, the local permutation test maintains the relation between the target and source histories, by decoupling only the source histories from the target events (Shorten et al., 2021). Nevertheless, to test this approach in comparison with established methods for the generation of surrogate data, we also implemented the algorithm based on random shuffling of the inter-event intervals, which preserves the probability distribution of the series of inter-event intervals; the iterative amplitude-adjusted Fourier transform (IAAFT) procedure (Schreiber and Schmitz, 1996; Perinelli et al., 2020), which preserves both distribution and power spectrum of the intervals; and the JODI algorithm (Ricci et al., 2019; Perinelli et al., 2020), which is specifically designed to preserve amplitude distribution and inter-event autocorrelation in point process data.

In all simulations and real data analyses, we implemented the nearest neighbor entropy estimator by using *k* = 30 neighbors and the maximum norm to compute distances (Faes et al., 2015), and generating a number of random time points equal to the number of target events (*N*_*U*_ = *N*_*X*_) (Mijatovic et al., 2021a). Analyses were repeated varying the length of the history embedding in the range *l* ∈ {1, 2, 3, 4, 5}. In the simulation study, the dependence of MIR and cMIR on the coupling parameter, type of distribution of the inter-event intervals, and time series length was also analyzed.

## 3 Simulation Study

This section reports the application of the proposed method for continuous-time estimation of the MIR on point processes simulated according to three scenarios. The first is devised to assess the bias of the MIR estimate on pairs of independent point processes for different types of inter-event distribution and distribution parameters. In the second and third simulation, coupled point processes designed to mimic the conditions of the real-data application relevant to cardiovascular variability reported in Section 4 are considered; specifically, the dynamics of the heartbeat times and of the arrival times of the blood pressure wave in the body periphery are reproduced, and the two processes are coupled in a way such that the intensity of their interaction increases or decreases depending on different driving mechanisms modulated by the input simulation parameter.

### 3.1 Simulation Design

#### 3.1.1 Simulation 1

In the first simulation, we generate pairs of uncoupled point processes according to different distributions. We consider: 1) Poisson processes, for which the inter-event intervals are i. i.d. exponential random variables with mean 1/*λ*_*P*_, where *λ*_*P*_ is the mean event-rate, here varied in the set *λ*_*P*_ ∈ {1, 2, 3, 4, 5} events/s; 2) point processes with i. i.d. inter-event intervals taken from the Gaussian distribution $N\left(\mu ,\sigma^{2}\right)$ , with mean varied in the set *μ* ∈ {0.8, 0.9, 1.0, 1.1, 1.2} s and standard deviation varied in the set *σ* ∈ {0.2, 0.4, 0.6, 0.8, 1.0} s; 3) point processes with i. i.d. inter-event intervals taken from the inverse Gaussian distribution IG (*μ*, *λ*), with mean varied in the set *μ* ∈ {0.8, 0.9, 1.0, 1.1, 1.2} s and shape parameter varied in the set *λ* ∈ {500, 600, 700, 800, 900} s; 4) point processes with identically distributed history-dependent inter-event intervals taken from the inverse Gaussian distribution, HDIG (*μ*, *λ*, *θ*), where *μ* ∈ {0.8, 0.9, 1.0, 1.1, 1.2} and *λ* ∈ {500, 600, 700, 800, 900} are the mean and shape parameters of an IG distribution, respectively, and *θ* is a vector of parameters that sets the correlations between the inter-event intervals of each process, and makes them it history-dependent (the values of the parameters in *θ* are described in Section 3.1.2).

While the first two distributions are typically used in the simulation of point processes, the IG and HDIG distributions are considered as they constitute the basis for a model that reproduces realistic heartbeat dynamics as presented in the following Section 3.1.2. For all these classes of point processes, the ground truth value of the information exchanged dynamically between the two processes is zero $\left({\dot{I}}_{X;Y}={\dot{T}}_{X\to Y}={\dot{T}}_{Y\to X}=0\right)$ because the processes are obtained from independent runs of the simulation. This allows to quantify the bias of the adopted MIR estimator, which is equivalent to the median value of the MIR estimated across several realizations of each simulation. In a single simulation, 100 realizations of each pair of uncoupled point processes were generated, each consisting of *N* = 300 events, and the distribution of the MIR measure was computed for each combination of the simulation parameters in the four cases described above.

#### 3.1.2 Simulation 2

In the second simulation the process *X*, which reproduces the heartbeat times, is generated as a point process following the history-dependent inverse Gaussian (HDIG) model proposed by Barbieri et al. (2005). According to this model, given any event *x*_*i*_ that simulates the occurrence time of a heartbeat, the waiting time until the next event, i.e. the *i*th inter-event interval *w*_*i*_, is assumed to be drawn from the probability density function

$$p\left(w_{i},X_{x_{i}}^{p},\theta ,\lambda \right)=\sqrt{\frac{\lambda }{2\pi w_{i}^{3}}}\cdot e^{\frac{-\lambda {\left[w_{i}-\mu \left(X_{x_{i}}^{p},\theta \right)\right]}^{2}}{2\mu {\left(X_{x_{i}}^{p},\theta \right)}^{2}w_{i}}},$$ (13)

where $\mu \left(X_{x_{i}}^{p},\theta \right)$ and *λ* are the mean and the scale parameter of the inverse Gaussian distribution. In the HDIG model, the mean is dependent on the history of the inter-event intervals up to the current event *x*_*i*_, $X_{x_{i}}^{p}=\left[w_{i-1},\ldots ,w_{i-p}\right]$ , according to the linear autoregressive (AR) model:

$$\mu \left(X_{x_{i}}^{p},\theta \right)=\theta_{0}+\overset{p}{\underset{j=1}{\sum }}\theta_{j}w_{i-j}.$$ (14)

This model represents, through the parameter vector *θ* = (*θ*_0_, *θ*_1_, … , *θ*_*p*_), the dependence of the present inter-event interval on the past history of the process, and in this application accounts for autonomic influences on heart rate variability (Stein et al., 1994). The setting of the model parameters is performed to reproduce typical point-process patterns of heart rate variability and cardiovascular interactions (Faes et al., 2014; Beda et al., 2017). Specifically, in our simulation we assume that the inter-event intervals exhibit lagged dependencies up to the order *p* = 5, and we set the coefficients {*θ*_1_, … , *θ*_5_} to obtain oscillations of *w*_*i*_ within the very low frequency (VLF, <0.04 Hz), low frequency (LF, 0.04–0.15 Hz) and high frequency (HF, 0.15–0.4 Hz) bands, as typically observed in the time series of heart period variability (Stein et al., 1994). This is achieved by simulating for the AR model (14) a transfer function with two complex-conjugate poles with modulus *ρ*_LF_ = 0.8 and phases ± 2*π* ⋅ 0.1 rad, two other complex-conjugate poles with modulus *ρ*_HF_ = 0.92 and phases ± 2*π* ⋅ 0.25 rad, and a real pole with modulus *ρ*_VLF_ = 0.6 (Beda et al., 2017). The mean and scale parameters of the inverse Gaussian distribution are set to *θ*_0_ = 1 s (average heart period) and *λ* = 600 s.

After generating the heartbeat point process *X* as described above, the point process *Y* that simulates the blood pressure arrival times is obtained generating its events as

$$y_{i}=x_{i}+\tau_{i},$$ (15)

where each propagation delay *τ*_*i*_ simulates an instance of the pulse arrival time (PAT), i.e. the time interval between the initiation of a cardiac contraction (identified by the electrical depolarization of the ventricles) and the following time of arrival of the blood pressure wave at the body periphery (identified by the time of maximum finger arterial pressure). The propagation delays are modelled as realizations of a second-order AR process defined as

$$\tau_{i}=a_{0}+a_{1}\tau_{i-1}+a_{2}\tau_{i-2}+u_{i},$$ (16)

where *a*_0_ represents the mean PAT set to 300 ms to reproduce the average propagation time of the sphygmic wave from the heart to the body periphery; *a*_1_ and *a*_2_ were set to reproduce a stochastic oscillation at ∼ 0.1 Hz by using a transfer function with two complex-conjugate poles with modulus *ρ*_LF_ = 0.8 and phases ± 2*π* ⋅ 0.1 rad, and *u*_*i*_ are random numbers taken from a Gaussian distribution with zero mean. The standard deviation of *u*_*i*_ was adjusted to obtain specific values for the standard deviation of *τ*_*i*_, which we denote as *σ*_PAT_. This important parameter modulates the variability of the arrival times *y*_*i*_, and in this simulation is inversely related to the strength of the interaction from *X* to *Y*; here, *σ*_PAT_ was varied from 10 to 235 ms with steps of 25 ms.

**FIGURE 2:**
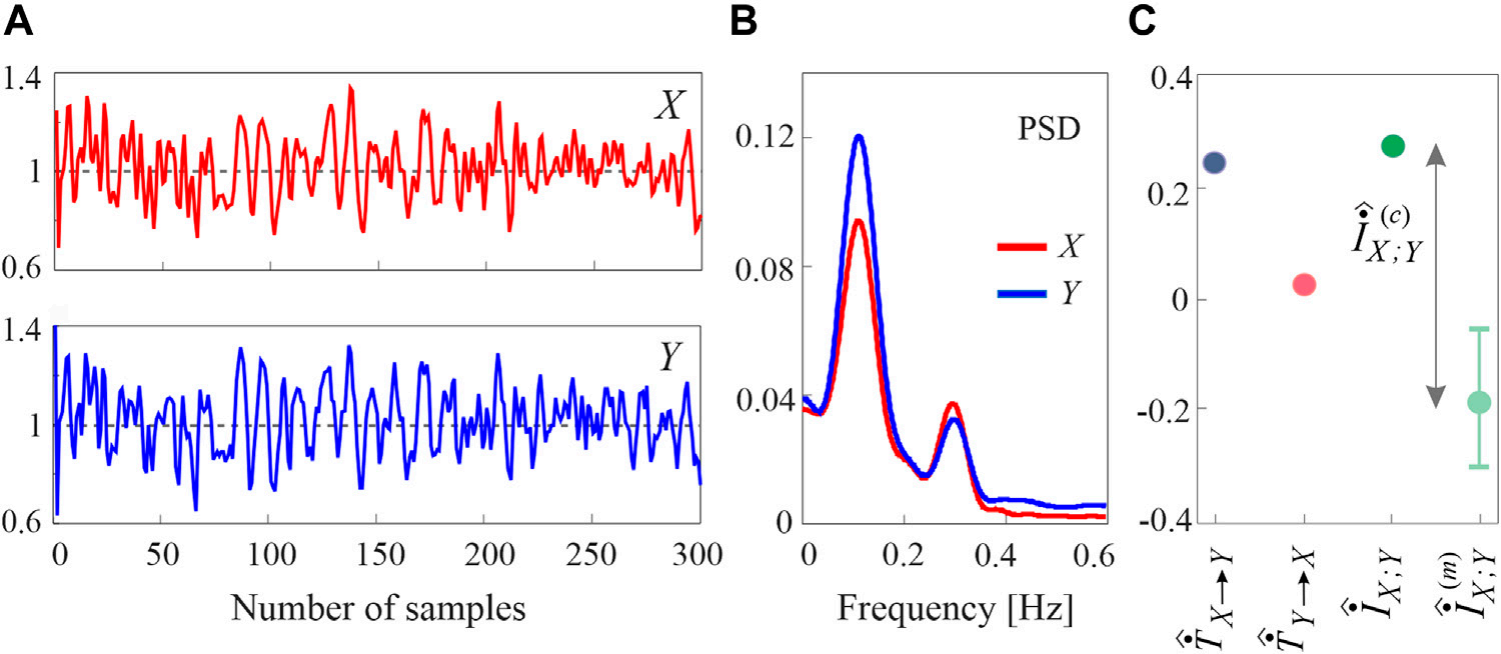
Representative example of the analysis relevant to the second simulation. **(A)** Inter-event intervals obtained for the process *X* as observations of the history-dependent inverse Gaussian model of Eqs. 13, 14 and for the process *Y* as observations of the process described by Eqs. 15, 16 generated with *σ*_PAT_ =85 ms; **(B)** power spectral densities of the two inter-event series evidencing LF and HF oscillations at ∼ 0.1 Hz and ∼0.25 Hz; **(C)** corresponding estimates of the TER from *X* to *Y* (blue circle) and from *Y* to *X* (red circle), of the MIR obtained as the sum of the two TERs (green circle), and distribution (median and percentiles) of the MIR estimated from 100 surrogate event series (light green); the difference between the MIR and the median of its distribution on the surrogates corresponds to the bias-corrected cMIR (gray arrow).

The inter-event intervals of the simulated heartbeat and blood pressure timings generated by a run of the simulation 2 are reported in Figure 2A) along with the respective power spectral densities (PSD, shown in Figure 2B), which evidence VLF, LF and HF oscillations in the two processes. The values of the TER estimated along the two directions of interaction, the MIR estimated as the sum of the two TERs, as well as the distribution of the MIR estimated from 100 surrogate series and the corresponding cMIR, are displayed in Figure 2C).

#### 3.1.3 Simulation 3

The third simulation is a modification of the second one and is devised to impose a common oscillation in the inter-event intervals of the process *X* and in the propagation delays *τ*_*i*_, so as to reproduce a condition in which the same underlying mechanism drives the two point processes. To this end, the HDIG model is retained to simulate the heartbeat intervals as in Eq. 13, but with different autocorrelation structure; specifically, an AR model of order *p* = 3 was used in Eq. 14, with coefficients {*θ*_1_, *θ*_2_, *θ*_3_} set to obtain oscillatory activity within the VLF and HF bands only (i.e., using a transfer function with one real pole with modulus *ρ*_HF_ = 0.92 and two complex conjugate poles with modulus *ρ*_HF_ = 0.92 and phases ± 2*π* ⋅ 0.25 rad). Starting from the intervals *w*_*i*_ drawn from this HDIG distribution with VLF and HF components, the LF component is introduced by adding to *w*_*i*_ a term equal to 2*τ*_*i*_, where *τ*_*i*_ is the random interval generated by Eq. 16. The simulation is then completed as in the previous case, i.e., by generating blood pressure arrival times as in Eq. 15 with propagation delays given again by Eq. 16. In this way, the LF component of the inter-event intervals in *X* and the propagation delays that contribute to the LF variability of *Y* are generated from the same random seed *u*_*i*_ and, as a consequence, the parameter *σ*_PAT_ that determines the variability of both components directly modulates the coupling between the two processes (i.e., we expect that higher values of *σ*_PAT_ determine higher amounts of information shared between *X* and *Y*).

### 3.2 Simulation Results

**FIGURE 3:**
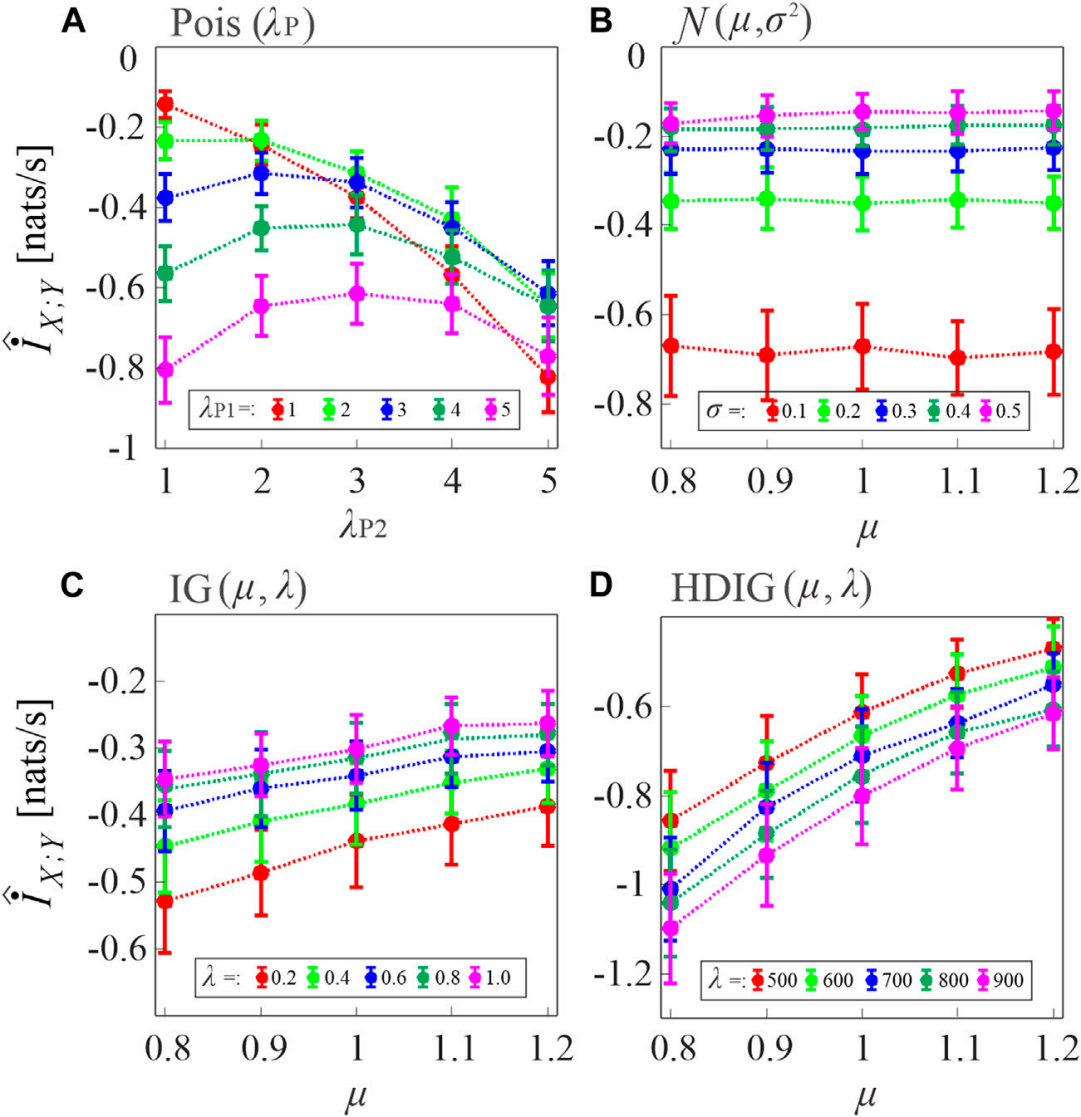
Assessment of the bias of the proposed MIR estimator. Plots depict the distribution (mean ± SD) of the MIR values computed over 100 realizations of uncoupled, short-length processes (*N* = 300 samples) with inter-event intervals taken from an exponential distribution with parameter *λ* (**(A)**, Poisson processes), a Gaussian distribution with mean *μ* and variance *σ* ^2^ **(B)**, an inverse Gaussian (IG) distribution with mean *μ* and shape parameter *λ* **(C)**, and a history-dependent inverse Gaussian (HDIG) distribution with mean *μ* and shape parameter *λ* **(D)**. The history embedding length was set to *l* = 1 in all computations.

In the first simulation, the MIR computed according to Eq. 8, where the two TER terms are estimated as in Eq. 11, was evaluated in pairs of uncoupled point processes by varying the type of inter-event interval distribution of the processes and the distribution parameters. Since for these processes the true value of the index is ${\dot{I}}_{X;Y}=0$ , the values of the MIR estimate ${\hat{\dot{I}}}_{X;Y}$ highlight the bias of the estimator. The results reported in Figure 3 indicate the presence of a negative bias in all simulations, as documented by the negative values of ${\hat{\dot{I}}}_{X;Y}$ measured by varying the type and parameters of the distribution of the uncoupled processes.

For Poisson processes, the bias tends to increase with the event rate and with the mismatch between the rates of the two processes (Figure 3A). For Gaussian processes, the bias increases when the standard deviation of the inter-event intervals is decreased, and is not substantially affected by the mean (Figure 3B). In the case of uncorrelated inverse Gaussian inter-event intervals, the bias is inversely related both to the mean and to the shape parameter of the interval distribution (Figure 3C); the dependence on the shape parameter becomes direct when the inverse Gaussian intervals are correlated in HDIG processes (Figure 3D). Overall, these results indicate that, in the presence of short realizations of point processes as in the present case where *N* = 300 spikes are simulated, the MIR estimates are strongly biased, and therefore strategies are needed for the compensation of such bias in the practical analysis of the information shared between point processes.

**FIGURE 4:**
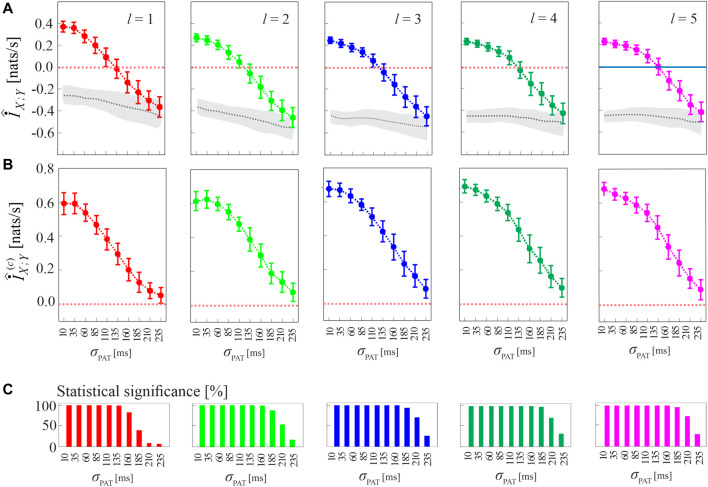
Computation of MIR and cMIR, and assessment of their statistical significance, in simulations of short-length (*N* =300 events) coupled history-dependent inverse Gaussian (HDIG) processes. **(A)** Distribution (mean ± SD) of the MIR measure, estimated over 100 realizations of simulation 2 as a function of the de-coupling parameter *σ*_PAT_, for different values of the history embedding length, *l* ∈ [1, 5]; gray dotted lines and shades correspond to the median and 5^*th*^ − 95^*th*^ percentiles of the distribution over the 100 realizations of the median MIR (for each realization, the median is computed over 100 surrogate pairs obtained using local permutation). **(B)** Distribution (mean ± SD) of the corrected MIR (cMIR) measure, estimated over the same realizations of simulation 2; for each realization, cMIR is computed as the difference between MIR and the median of the MIR distribution assessed over 100 local permutation surrogates. **(C)** Bar plots reporting the number of realizations for which the MIR is detected as statistically significant according to the surrogate data analysis.

The procedure for compensating the bias of MIR estimates, as well as the performance of the corrected cMIR estimator, are illustrated in Figure 4 for several runs of simulation 2 generated by varying the intensity of the interaction between the HDIG processes modulated by the parameter *σ*_PAT_.

As shown in Figure 4A, the progressive de-coupling of the interactions between *X* and *Y* obtained by increasing *σ*_PAT_ is reflected by a progressive decrease of the MIR estimates; this behavior is observed for all the analyzed values of the history embedding length *l*. However, the analysis also confirms the presence of a substantial bias in the estimates of MIR, which take on negative values when the coupling between the two processes decreases. Figure 4B reports the bias-corrected MIR estimates, showing how the correction leads to non-negative values of cMIR even when the processes approach the uncoupled states for high values of *σ*_PAT_. The correction brings the cMIR values in the range 0 − 0.6 nats/s for *l* = 1, which extends to ∼ 0.7 nats/s for *l* = 5, and evidences the appropriateness of using higher embedding lengths in the simulated process when the inter-event intervals are modeled by an AR model of order *p* = 5.

The benefit of longer history embeddings is documented also in Figure 4C, where we employ the standard procedure for testing coupling significance based on surrogate data. This procedure tests the null hypothesis of uncoupling between the two analyzed point processes and is based on generating, from each pair of original realizations of the processes, a suitable number of pairs of surrogate event series using the local permutation method, and then on deeming the original pair as significantly coupled if the MIR value was above the 95th percentile of the MIR surrogate distribution. The percentage of realizations for which the MIR/cMIR values were detected as statistically significant is reported in Figure 4C, showing that the rate of detection of weakly coupled point processes (higher values of *σ*_PAT_) increases for higher embedding lengths.

**FIGURE 5:**
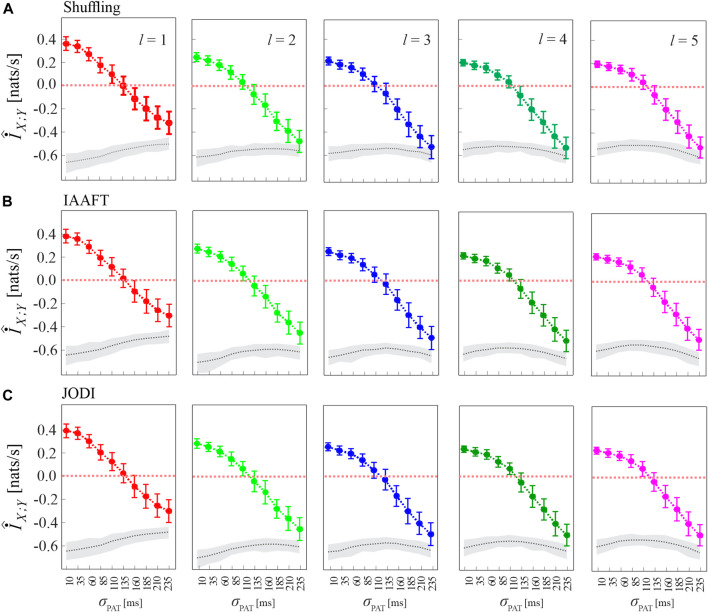
Analysis of MIR using different methods for surrogate data generation. Plots depict the distribution (mean ± SD) of the MIR measure, estimated over 100 realizations of simulation 2 as a function of the de-coupling parameter *σ*_PAT_, for different values of the history embedding length, *l* ∈ [1, 5]. Gray dotted lines and shades correspond to the median and 5^*th*^ − 95^*th*^ percentiles of the distribution over the 100 realizations of the median MIR; for each realization, the median is computed over 100 surrogate pairs generated by random shuffling of the inter-event intervals **(A)**, according to the IAAFT algorithm **(B)**, and according to the JODI algorithm **(C)**.

Figure 5 has the same structure of Figure 4A, and shows alternative approaches to generate the surrogate data consistent with the null hypothesis of uncoupling between the two analyzed point processes. The figure shows that the analysis of cMIR is rather stable at varying the type of surrogate data. The most remarkable difference is that using the shuffling surrogates, similarly to the local permutation surrogates employed in Figure 4A even though with a lower extent, the MIR estimates partially overlap with the distribution of the MIR for the original process realizations when the de-coupling parameter is high (*σ*_PAT_ = 210 ms and particularly *σ*_PAT_ = 235 ms); such an effect is not observed using IAAFT and JODI surrogates. This suggests that surrogates which preserve autocorrelation properties of the inter-event intervals are more prone to detect weak but significant amounts of information shared by two point processes and to return higher values of the cMIR measure. On the other hand, the use of the local permutation method for the generation of surrogate time series resulted in higher values of the MIR assessed on the surrogates (see the gray areas in Figure 4A vs those in Figure 5). This result is expected, as the local permutation method maintains the relationship of the source history embeddings with the history embeddings of the target, thus allowing to keep a low rate of false positive detection of information transfer (Shorten et al., 2021). Thus the comparison between Figures 4A, 5 evidences that the local permutation surrogates adopted as a main solution in our work tends to favor specificity in the detection of coupled point process dynamics, while surrogates preserving autocorrelation structure of the inter-event intervals tend to favor sensitivity. These considerations are of practical relevance for the analysis of real-world data.

**FIGURE 6:**
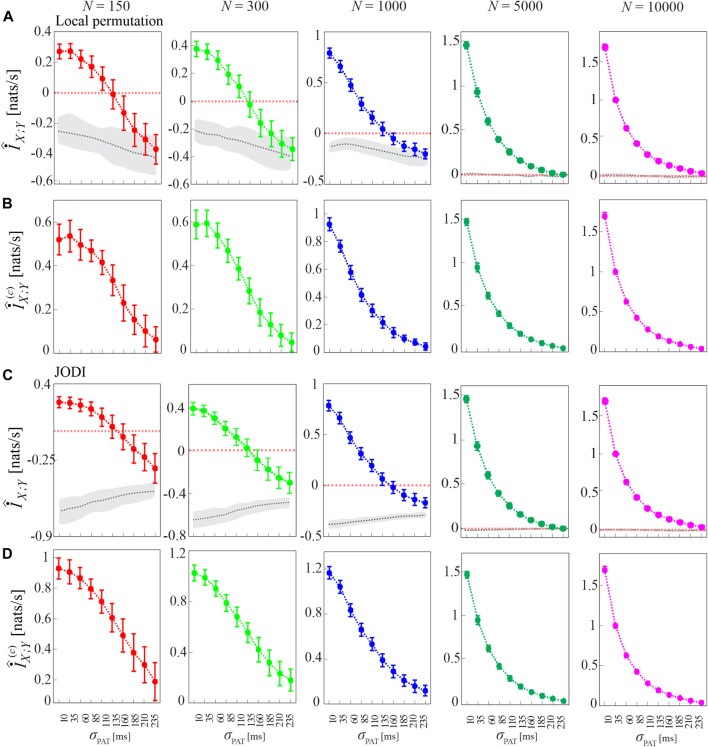
Dependence of MIR and cMIR on the size of the analyzed point processes and on the type of surrogate series used for bias compensation. Plots depict the distribution (mean ± SD) of the MIR **(A,C)**, and the cMIR based on local permutation surrogates **(B)** or JODI surrogates **(D)**, estimated (history embedding length *l* = 1) as a function of the decoupling parameter *σ*_PAT_ over 100 realizations of simulation 2 of different lengths (number of simulated events *N* ∈ {150, 300, 1000, 5000, 10 000}). In panels **(A,C)**, gray dotted lines and shades correspond to the median and 5^*th*^ − 95^*th*^ percentiles of the distribution over the 100 realizations of the median MIR, where for each realization the median is computed over 100 surrogate pairs obtained by using local permutation surrogates **(A)** or JODI surrogates **(C)**.

To show that the bias of the MIR estimates is due to the small sample size of the point process realizations analyzed, in Figure 6 we show the MIR computed as a function of the decoupling parameter *σ*_PAT_ for different lengths of the simulated processes, *N* ∈ {150, 300, 1,000, 5,000, 10,000}, together with the cMIR obtained using either the local permutation method or the JODI algorithm to generate surrogate point processes. We observe that increasing the number of simulated events progressively reduces the bias, as documented by the progressively higher values observed for the MIR and by the absence of negative values for *N* ≥ 5,000. As expected, also the variance of the MIR estimates decreases while increasing *N*, confirming that larger sample sizes reduce not only the bias, but also the variability of the estimates. We also note that the median of MIR over the surrogate distribution (gray dotted line in Figures 6A,C) is not a constant function of *σ*_PAT_ and differs for the two methods for surrogate generation. As a consequence, the cMIR does not represent a simple translation of MIR toward positive values and depends on the adopted surrogates. In particular, the use of local permutation surrogates results in lower values of cMIR compared to that based on JODI surrogates (see Figures 6B,D), suggesting a better bias compensation for the latter approach. Moreover, the non-monotonic behavior of MIR estimated for small sample size (*N* = 150 and *N* = 300) is accentuated in cMIR when local permutation surrogates are used (Figures 6A,B), while it is smoothed when JODI surrogates are used (Figures 6C,D).

**FIGURE 7:**
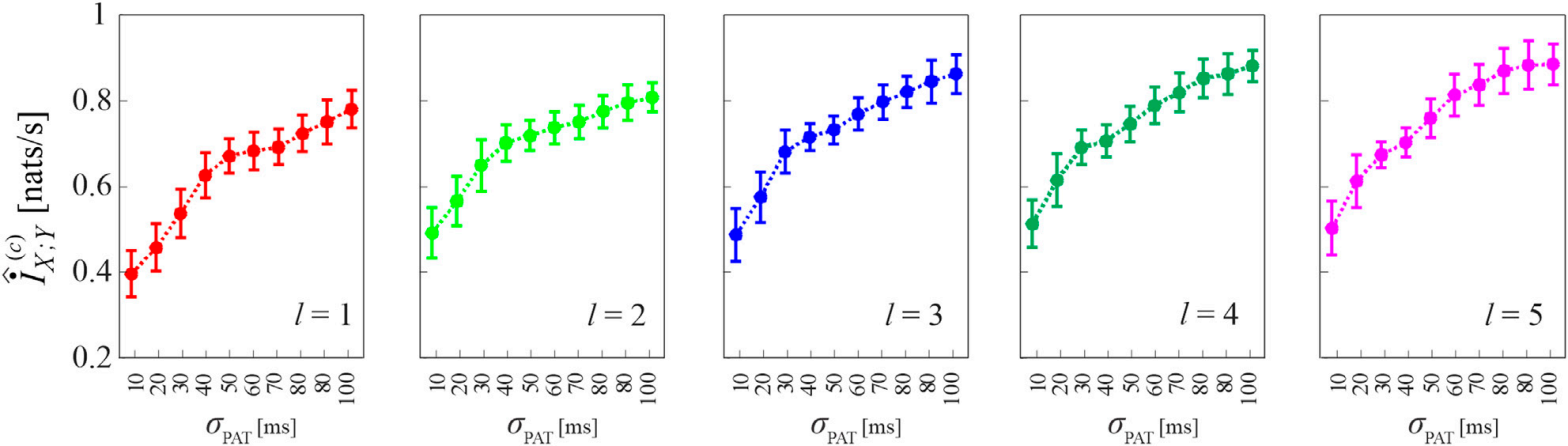
Computation of cMIR for short-length realizations (*N* =300 events) of simulation 3. Plots depict the distribution (mean ± SD) of the cMIR measure, estimated over 100 realizations of simulation 3 as a function of the parameter *σ*_PAT_, for different values of the history embedding length, *l* ∈[1,5]. Note that in this simulation in which common oscillations are imposed in the variability of the inter-event intervals of the process *X* and on the propagation delay from *X* to *Y*, *σ*_PAT_ serves as a coupling parameter.

Figure 7 reports the results of Simulation 3, where coupled HDIG processes are generated so that increasing the variability of the propagation delay from *X* to *Y* may also increase the coupling between the two processes. This effect is verified in our simulations by observing that the cMIR measure increases with the parameter *σ*_PAT_, which in this case modulates the variability of both the LF component of the inter-event intervals of *X* and the propagation delays; the increase of the information shared between the two processes at increasing *σ*_PAT_ is observed consistently for all the analyzed history embedding lengths, *l* ∈ [1, 5].

## 4 Application to Real Data

This section describes the application of cMIR on experimental point-process data relevant to cardiovascular variability. In the information-theoretic domain, cardiovascular interactions are commonly studied by means of entropy measures applied to the discrete-time series of heart period and arterial pressure variability (Faes et al., 2012; Faes et al., 2015; Javorka et al., 2017). However, given the intrinsic unevenly sampled nature of human heartbeats (Barbieri et al., 2005), recent studies started to face the analysis of cardiovascular, cardiorespiratory and brain-heart dynamics from the perspective of point processes analyzed also using information measures (Valenza et al., 2018; Greco et al., 2019). Here, with the aim of assessing the potential of MIR analysis in short-term cardiovascular variability as well physiological mechanisms other than those investigated by the traditional information-theoretic measures, we apply our continuous-time approach on the point processes that map the heartbeat and systolic time events measured in healthy humans and monitored under different physiological states.

### 4.1 Database and Experimental Protocol

The analyzed data belong to an historical database previously used to study the effects of physiological stress and cognitive workload on cardiovascular variability (Javorka et al., 2017; Pernice et al., 2019). The data were acquired on 76 young healthy subjects (age: 18.4 ± 2.7 years, 32 males), normotensive and with a normal body mass index (21.3 ± 2.3 kg/m^2^), and consisted of electrocardiographic (ECG) and blood pressure (BP) recordings acquired synchronously with a sampling frequency of 1 kHz. ECG and BP signals were recorded by using CardioFax ECG-9620 (Nihon Kohden, Japan; horizontal bipolar thoracic leads) and the Finometer Pro devices (FMS, Netherlands; volume-clamp continuous BP measurement), respectively. The experimental protocol foresaw the acquisition of the signals in different physiological states, going from resting conditions to different types of stress (orthostatic or mental). For the analyses carried out in this work, we have taken into account the following states: 1) baseline state (B), with subjects resting in the supine position for 15 min; 2) head-up tilt state (T), obtained by passively tilting the subjects by 45° to the upright position and maintaining them in that state for 8 min in order to produce orthostatic stress; 3) mental arithmetic state (M), obtained with subjects in the supine position and by asking them to sum up as fast as possible 3-digit numbers projected on the ceiling until reaching a 1-digit number and to decide whether the resulting number was even or odd (PMT test, Psycho Soft Software, s. r.o., Brno, Czech Republic), where this task was repeated over a period of 6 min to elicit cognitive load. Further details on the experimental protocol can be found in (Javorka et al., 2017; Pernice et al., 2019).

### 4.2 Data Analysis

The data analyzed consisted of sequences containing the timings of the consecutive R peaks in the ECG (event series of the R times) and of the following maxima in the BP signals (event series of the systolic times), previously extracted by means of LabChart 8 (ECG analysis, blood pressure modules) toolbox from ADInstruments (Javorka et al., 2017; Pernice et al., 2019). Moreover, the time series of the RR and PAT intervals were measured respectively as the sequences of the difference between two consecutive R times, and of the difference between each systolic time and the preceding R time. The event series and time series analyzed for each subject and experimental condition consisted of *N* = 300 events, which were extracted starting respectively ∼8 min after the beginning of the phase B, ∼3 min after the beginning of the phase T, and ∼2 min after the beginning of the phase M; the corresponding RR and PAT time series were checked for stationarity by using a test targeting a restricted form of weak stationarity (Magagnin et al., 2011).

Starting from the interval series of RR and PAT, the mean and standard deviation of the two series, respectively computed as the average interval duration and the interval variability, were computed for each subject and experimental condition. Starting from the corresponding event series of R times and systolic times, the intervals forming the history embeddings were extracted as displayed in Figure 1, and employed as described in Section 2 to estimate first the TER along the two directions of interaction, then the MIR, and finally the cMIR. To test the statistical significance of the differences in the median of the distributions of each measure (mean, standard deviation and cMIR) evaluated across conditions (B, T, M), we used the non-parametric Kruskal-Wallis test, followed by post-hoc paired Wilcoxon signed rank test to assess pairwise differences (B vs. T, B vs. M, T vs. M) with 5% significance and employing the Bonferroni-Holm correction for multiple comparisons.

### 4.3 Results and Discussion

**FIGURE 8:**
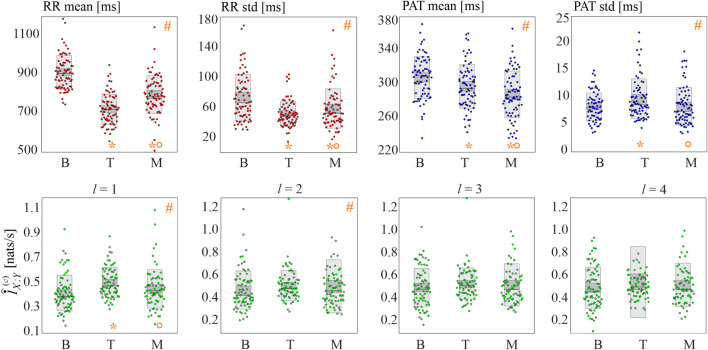
Basic statistics and information shared in the cardiovascular time series. Panels depict the boxplots and individual values of the mean and standard deviation of the RR intervals (red dots) and of the PAT intervals (blue dots), as well as of the cMIR measure estimated for different values of the history embedding length *l* (green dots), computed for all subjects during the three analyzed experimental conditions (baseline (B), head-up tilt (T), and mental arithmetic (M)). Statistical analysis (orange symbols): *#*, *p* < 0.05, Kruskal-Wallis test; *p < 0.05 B vs T or B vs M; °*p* < 0.05: T vs M, Wilcoxon test.

The results of the real data analysis are summarized in Figure 8, reporting the distributions of the basic statistics (mean and standard deviation of RR and PAT intervals) in the upper panels and of cMIR in the lower panels.

The mean RR interval decreased significantly moving from B to T and from B to M; the effect was more pronounced during tilt than during mental arithmetic. Similarly, both postural stress and mental stress induced a decrease of the variability of the RR intervals, with a larger effect during head-up tilt, as documented by the statistically significant decrease of the standard deviation of the RR intervals moving from B to T and from B to M and by its higher values during M compared to T.

The physiological stressors induced also statistically significant variations in the mean and variability of the propagation delays of the sphygmic wave from the heart to the periphery. Specifically, the mean PAT decreased progressively and significantly while moving from B to T and from T to M, and the standard deviation of PAT increased during T compared to B, and decreased during M compared to T.

The analysis of the cMIR measure indicated that the postural stress tends to increase the information shared between the R times and the systolic times, while mental stress does not have significant effects. In fact, cMIR was significantly higher during T compared to B, and significantly lower during M compared to T when a history embedding *l* = 1 was used. These variations were less evident when *l* = 2, as the Kruskal-Wallis test reported statistically significant differences among the three distributions despite the post-hoc tests did not reach statistical significance (B vs T, *p* = 0.070; B vs M, *p* = 0.195, T vs M, *p* = 0.333), and were reduced to non-significant trends when *l* = 3 and *l* = 4.

The alterations observed in the basic cardiovascular parameters during the two physiological stressors are in agreement with a large body of literature in cardiovascular variability analysis, and document the involvement of several physiological mechanisms in the elicitation of these stressors. In particular, the lower mean and variability of the RR intervals during tilt and mental arithmetic reflect well-known effects such as the tachycardia and the shift of the cardiac autonomic balance towards sympathetic activation and parasympathetic inhibition induced by postural and mental stress (Montano et al., 1994; Carnethon et al., 2002; Garde et al., 2002; Wood et al., 2002; Martinelli et al., 2005; Javorka et al., 2017, 2018; Kim et al., 2018; Pernice et al., 2019). The interpretation of the shortening of PAT and of the increase of its variability observed during tilt is less straightforward. The PAT is composed by the pre-ejection period (PEP), i.e., the interval from the electrical depolarization of the ventricles to the ejection of the blood from the heart, and by the pulse transit time (PTT), i.e., the time that it takes for the blood pressure wave to reach the body periphery; the PEP depends mainly on the strength of left ventricular contraction, influenced by the Frank-Starling law and by sympathetic control (Krohová et al., 2017), while the PTT is mostly affected by arterial compliance, reflecting (on a short time scale) modulation of blood pressure and vasomotion (Mukkamala et al., 2015; Czippelova et al., 2019). In accordance with our previous research in a related database (Krohová et al., 2017), we expect an increase in PEP during orthostasis as an effect of decreased diastolic filling of the heart via the Frank-Starling mechanism leading to a lower strength of the cardiac contraction. Therefore, the decrease of the mean PAT observed during tilt should reflect mostly a decrease in PTT related to an augmented arterial stiffness caused by peripheral vasoconstriction, which is in turn evoked by the vascular baroreflex response associated with a decrease of blood pressure due to pooling of blood in the lower extremities (Czippelova et al., 2019); the concomitance of these opposite trends (i.e., increase of PEP and decrease of PTT) and the complexity of the related physiological mechanisms including autonomic reflexes and mechanical effects (Rapalis et al., 2017; Czippelova et al., 2019; Pernice et al., 2021) may - together with an increased systolic blood pressure variability associated with tilt - explain the higher variability of PAT observed during tilt. During cognitive load, induced in our protocol by the mental arithmetic task, the more prominent decrease of PAT likely reflects–in addition to vasoconstriction driven by commands stemming from the central nervous system which reduces the PTT–also a reduction of PEP associated with an increased cardiac contractility mediated by the sympathetic nervous system (Martin et al., 2016); in this case, the presence of common trends (i.e., decrease of both PEP and PTT) may explain both the lower PAT and its lower variability measured during mental arithmetic.

According to our results, the physiological mechanisms described above are associated with an increase of the rate of information exchange between the point processes marking the R times of the ECG and the times of arrival of the sphygmic wave in the body periphery. Higher values of MIR are expected when the variations of the propagation delay from one process to another are small, or when such variations occur in phase due to the effect of some common driver mechanism. Since we observe an increase in cMIR simultaneously with a shortening of the mean PAT and an increase of the PAT variability, we conclude that the presence of a common driver oscillation is the mechanism underlying the higher exchange of information. This mechanism was synthetically reproduced in our third simulation (see Figure 7), and can be physiologically explained by the sympathetic activation induced by head-up tilt (Montano et al., 1994; Carnethon et al., 2002; Martinelli et al., 2005). The “common driver” nature of this mechanism can be explained by observing that during postural stress the sympathetic activation is related to the baroreflex mechanism and, as such, it simultaneously involves the variability of the heart period (and thus that of the R times) and the variability of the arterial pressure (and thus that of the PAT) (Porta et al., 2011; Faes et al., 2013), thereby determining a more intense exchange of information between the two processes. In fact, vasoconstriction in the arterioles in systemic circulation is modulated almost exclusively by the sympathetic part of the autonomic nervous system (Krohova et al., 2020) whose oscillations mostly occur in the LF band; a similar effect is mimicked in our simulations in Section 3. On the other hand, the less evident variations of cMIR observed during the mental arithmetic test may be associated with the fact that the sympathetic activation evoked by mental stress is of a different type, likely involving central commands from the upper brain centers (cortex) which control more independently the heartbeat and the arterial compliance without prominent synchronization effects related to the baroreflex (Fauvel et al., 2000).

The observation of statistically significant differences across conditions of the cMIR index only for small values of the history embedding length (variations from B to T and from T to M are detected for *l* = 1 and, to a lower extent, for *l* = 2) suggests that the cardiovascular interactions altered by physiological stress occur mostly as a consequence of the variability of the propagation time of the sphygmic wave from the heart to the body periphery, and that the use of longer memory effects may confound the detection of such altered interactions. This result can be expected by considering that the largest part of the analyzed type of interactions is due to the PAT, whose effects are fully captured with *l* = 1 (note that, within the point process framework, effects explained with *l* = 1 are not immediate but rather indicative of time-lagged effects with short memory). The result is in agreement with previous observations reporting that the latency of cardiovascular information transfer is typically limited to zero-lag or one-beat interactions, especially during postural stress (Faes et al., 2014). Nevertheless, we remark that the type of cardiovascular interactions studied using time-series based methods (Faes et al., 2014; Porta and Faes, 2015) reflect different mechanisms than those reflected by the event-based method employed here, the former being related mainly to the baroreflex control of heart rate, while the latter being related to blood pulse propagation and arterial contractility.

## 5 Concluding Remarks

This study reports the first application to cardiovascular dynamics of the continuous-time estimator of the information exchanged dynamically between point processes introduced in (Shorten et al., 2021) to compute the TER and employed in (Mijatovic et al., 2021a) to compute the MIR. In the reported application context where the direction of interaction is determined by the cardiac pacemaker that triggers the propagation of the sphygmic waves through the arterial bed, studying causal interactions through the TER is less relevant than assessing the coupling between the heartbeat and systolic times through the MIR. Moreover, this application context is particularly challenging with regard to the computation of information rates, because the cardiovascular regulation operates mostly through short-term control mechanisms and needs to be performed over short stationary series including a few hundred heartbeats at most (Cohen and Taylor, 2002). The adopted estimator combines the property that for point processes the MIR can be formulated in terms of the TER (Mijatovic et al., 2021a), and exploits the approach based on representing dynamic states of point processes in terms of inter-event intervals to efficiently capture information flows (Shorten et al., 2021). In this work we investigate the small sample properties of the MIR estimator, finding the presence of a negative bias which is significant in almost all the scenarios simulated between uncoupled point processes (Figure 3). A similar bias, even though considerably smaller, was described in the work that first introduced the TER and MIR estimators (Mijatovic et al., 2021a; Shorten et al., 2021). As opposed to previous applications in neuroscience, cardiovascular interactions feature conditions of strongly auto-correlated processes and of short data sequences, which can be responsible of the strong bias that very often leads to meaningless negative values of MIR, thus justifying the adoption of countermeasures to prevent such bias. In Shorten et al. (2021), this bias was associated with a violation of the assumption of local uniformity of the probability density within the range of the *k* nearest neighbors used for entropy estimation. While methods for reducing the bias of nearest neighbor information estimators which address specifically cases where local uniformity does not apply can be devised (Gao et al., 2015), in this work we resort to an empirical approach that reproduces the bias of MIR estimated over uncoupled surrogate time series, and then subtracts this bias from the MIR computed for the original series. This empirical approach has the advantage of generality, since surrogates mimic the data distribution and are in principle able to reproduce diverse sources of bias and to compensate them in the corrected measure (Papana et al., 2011). We find that different procedures for surrogate data generation have a different impact on the detection coupling and on the compensation of the bias, with differences being emphasized as the size of the analyzed event series decreases. A main advantage of the resulting cMIR measure is that it establishes the statistical significance of the information shared by the two processes, meaning that it does not indicate significant coupling when the coupling is indeed absent (Papana et al., 2011); this aspect has been verified in our simulations showing that cMIR tends to zero when the studied processes approach the uncoupled regime. A drawback of the proposed correction stands in the fact that it reproduces the bias for uncoupled processes, which can be different than that occurring in the case of coupling. As a consequence, since the MIR for truly coupled signals can be affected by a different bias than that observed for uncoupled signals, our approach does not provide a rigorous correction of the bias when the coupling is nonzero and some residual bias possibly remains also after the correction. Moreover, some applications of cMIR to networks with several connections to be estimated can become computationally unfeasible since the generation and information-theoretic analysis of surrogate point processes is a time-consuming procedure.

The proposed approach to estimate MIR in the presence of short and possibly noisy point process data is recommended for applications in the field of Network Physiology, where the estimation of organ system interactions is typically challenged by the inherently complex nature of human physiological signals (Lehnertz et al., 2020). In our work, where complex point process interactions between the heartbeat timings and the arrival times of the sphygmic wave on the body periphery have been analyzed, we detected significant coupling between the two processes in all subjects and experimental conditions. Moreover, the statistically significant variations of cMIR observed during physiological stress suggest that the index can reflect the neuroautonomic modulation of the heartbeat and vascular dynamics. This conclusion is supported by previous studies performed by using different approaches working in discrete time on interval time series, which suggests that the differences between heart rate and pulse rate variability are due not only to measurement noise, but also to physiological factors (Schäfer and Vagedes, 2013; Pernice et al., 2019). These factors are related to the physiological modulation of the two time intervals that compose the PAT, i.e. the PEP and the PTT. According to our present findings and previous research (Krohová et al., 2017; Czippelova et al., 2019; Pernice et al., 2019), the increased variability of PAT observed during postural stress arises from an increased variability of PEP related to sympathetic influence on cardiac contractility, an increased variability of systolic blood pressure leading to increased PTT variability, and an increased variability in the vascular tone related to sympathetic vasomotor control. These effects are manifested mainly in the LF band (0.04–0.15 Hz) of the spectrum, which is the frequency range where dominant oscillations of the blood pressure and the heart rate are observed during head-up tilt (Montano et al., 1994; Pernice et al., 2021). Accordingly, we ascribe the increase of cMIR observed during postural stress to the activation of the sympathetic nervous system and to the increased chronotropic baroreflex coupling occurring with tilt, which are likely responsible of the synchronous modulation of the LF variability of heart rate and PAT. Whilst we support this interpretation with our simulation, a recent study showed that heart rate and PAT variability are more correlated at the frequency of the Mayer waves (∼ 0.1 Hz) (Peng et al., 2021). On the other hand, the smaller changes of cMIR observed during mental arithmetic suggest that mental stress evokes a different type of sympathetic activation, possibly more of central origin than related to common modulation of heart rate and vascular tone (Javorka et al., 2017). Future studies should address the separate role of PEP and PTT variability in the changes of the coupling between heartbeat and systolic time dynamics, and investigate the clinical value (e.g., in relation to the alterations of the arterial compliance observed with aging or hypertension) of the novel measures computed in this work.

In summary, the method for MIR computation presented in this work constitutes a viable approach to assess the rate of information exchanged dynamically between pairs of point processes from short realizations of event-based data. Our approach, which explicitly considers the point-process structure of human heartbeats, is alternative to existing model-free information measures developed in discrete time and working on amplitudes rather than on events (Porta and Faes, 2015), as well as to existing model-based parametric models developed in the point process framework (Barbieri et al., 2005; Valenza et al., 2018; Greco et al., 2019). As such, it holds the potential to disclose different physiological mechanisms than those investigated by traditional cardiovascular variability approaches.

## Data Availability

The experimental data used for this article are available upon request to the corresponding Author. The software packages relevant to MIR and cMIR estimation, and to different algorithms for generation of surrogates of event sequences, are available for free download from the GitHub repositories https://github.com/mijatovicg/TEMI, and https://github.com/LeonardoRicci/SpiSeMe.

## Appendix

This section reports the derivation of the decomposition of the MIR presented in Eq. 6. We start considering two discrete-time processes $X_{\Delta }=\left\{X_{t_{n}}\right\}$ and $Y_{\Delta }=\left\{Y_{t_{n}}\right\}$ defined at the discrete time instants $t_{n}=n\Delta t,n\in Z$ , where Δ*t* is the time interval between samples expressed in unit of time; the derivation in continuous time, i.e., for Δ*t* → 0, will intuitively follow. In discrete time, the MIR is defined as

$${\dot{I}}_{X_{\Delta };Y_{\Delta }}=\underset{n\to \infty }{\lim}\frac{1}{n\Delta t}I\left(X_{t_{1}:t_{n}};Y_{t_{1}:t_{n}}\right),$$
(17)

where $X_{t_{1}:t_{n}}=\left\{X_{\Delta t},X_{2\Delta t},\ldots ,X_{n\Delta t}\right\}$ and $Y_{t_{1}:t_{n}}=\left\{Y_{\Delta t},Y_{2\Delta t},\ldots ,Y_{n\Delta t}\right\}$ are *n*-dimensional vectors of consecutive random variables taken from the two processes. According to basic information-theoretic rules we have that $I\left(X_{t_{1}:t_{n}};Y_{t_{1}:t_{n}}\right)=H\left(X_{t_{1}:t_{n}}\right)+H\left(Y_{t_{1}:t_{n}}\right)-H\left(X_{t_{1}:t_{n}},Y_{t_{1}:t_{n}}\right)$ , from which Eq. 17 can be expressed in terms of entropy rates as

$${\dot{I}}_{X_{\Delta };Y_{\Delta }}={\dot{H}}_{X_{\Delta }}+{\dot{H}}_{Y_{\Delta }}-{\dot{H}}_{X_{\Delta },Y_{\Delta }}.$$
(18)

Then, recalling the equivalent definitions of entropy rate for sequences of identically distributed random variables (Cover, 1999), the entropy rate for the process *X*_Δ_ can be written as

$${\dot{H}}_{X_{\Delta }}=\frac{1}{\Delta t}\underset{n\to \infty }{\lim}\frac{H\left(X_{t_{1}:t_{n}}\right)}{n}\equiv \frac{1}{\Delta t}\underset{n\to \infty }{\lim}H\left(X_{t_{i}}|X_{t_{i-n}:t_{i-1}}\right)=\frac{1}{\Delta t}H\left(X_{t_{i}}|X_{t_{i}}^{-}\right),$$
(19)

where the last term is independent on *t*_*i*_ due to stationarity and is written in compact form evidencing the past history of the process, $X_{t_{i}}^{-}=X_{t_{i-n}:t_{i-1}}$ with *n* → *∞*. By using Eqs. 18, 19 can be formulated evidencing a difference of MI terms as

$$\begin{array}{cc} {\dot{I}}_{X_{\Delta };Y_{\Delta }} & =\frac{1}{\Delta t}\left(H\left(X_{t_{i}}|X_{t_{i}}^{-}\right)+H\left(Y_{t_{i}}|Y_{t_{i}}^{-}\right)\right)-H\left(X_{t_{i}},Y_{t_{i}}|X_{t_{i}}^{-},Y_{t_{i}}^{-}\right)\left.\right)\\ & =\frac{1}{\Delta t}\left(H\left(X_{t_{i}},X_{t_{i}}^{-}\right)-H\left(X_{t_{i}}^{-}\right)+H\left(Y_{t_{i}},Y_{t_{i}}^{-}\right)-H\left(Y_{t_{i}}^{-}\right)-H\left(X_{t_{i}},Y_{t_{i}},X_{t_{i}}^{-},Y_{t_{i}}^{-}\right)+H\left(X_{t_{i}}^{-},Y_{t_{i}}^{-}\right)\right)\\ & =\frac{1}{\Delta t}\left(I\left(X_{t_{i}},X_{t_{i}}^{-};Y_{t_{i}},Y_{t_{i}}^{-}\right)-I\left(X_{t_{i}};Y_{t_{i}}^{-}\right)\right). \end{array}$$
(20)

Moreover, application of the chain rule for mutual information allows to expand the first MI term in the last line of Eq. 20 as

$$\begin{array}{cc} I\left(X_{t_{i}},X_{t_{i}}^{-};Y_{t_{i}},Y_{t_{i}}^{-}\right) & =I\left(X_{t_{i}}^{-};Y_{t_{i}},Y_{t_{i}}^{-}\right)+I\left(X_{t_{i}};Y_{t_{i}},Y_{t_{i}}^{-}|X_{t_{i}}^{-}\right)\\ & =I\left(X_{t_{i}}^{-};Y_{t_{i}}^{-}\right)+I\left(Y_{t_{i}},X_{t_{i}}^{-}|Y_{t_{i}}^{-}\right)+I\left(X_{t_{i}};Y_{t_{i}},Y_{t_{i}}^{-}|X_{t_{i}}^{-}\right)\\ & =I\left(X_{t_{i}}^{-};Y_{t_{i}}^{-}\right)+I\left(Y_{t_{i}},X_{t_{i}}^{-}|Y_{t_{i}}^{-}\right)+I\left(X_{t_{i}};Y_{t_{i}}^{-}|X_{t_{i}}^{-}\right)+I\left(X_{t_{i}};Y_{t_{i}}|X_{t_{i}}^{-},Y_{t_{i}}^{-}\right). \end{array}$$
(21)

Finally, substituting Eqs. 20, 21 leads to express the MIR as

$$\begin{array}{cc} {\dot{I}}_{X_{\Delta };Y_{\Delta }} & =\frac{1}{\Delta t}\left(I\left(Y_{t_{i}},X_{t_{i}}^{-}|Y_{t_{i}}^{-}\right)+I\left(X_{t_{i}};Y_{t_{i}}^{-}|X_{t_{i}}^{-}\right)+I\left(X_{t_{i}};Y_{t_{i}}|X_{t_{i}}^{-},Y_{t_{i}}^{-}\right)\right)\\ & =\frac{1}{\Delta t}\left(T_{X_{\Delta }\to Y_{\Delta }}+T_{Y_{\Delta }\to X_{\Delta }}+I_{X_{\Delta };Y_{\Delta }}^{0}\right), \end{array}$$
(22)

where the transfer entropies $T_{X_{\Delta }\to Y_{\Delta }}$ and $T_{Y_{\Delta }\to X_{\Delta }}$ (Schreiber, 2000) and the instantaneous information exchanged between the two processes, $I_{X_{\Delta };Y_{\Delta }}^{0}$ (Amblard and Michel, 2013), are put in evidence. Taking the limit Δ*t* → 0 in Eq. 22 leads to Eq. 6, which is valid when the processes *X* and *Y* are continuous.
